# Towards a deeper understanding of male involvement in the prevention of mother to child transmission of HIV in the Bogodogo District of the Central Region of Burkina Faso

**DOI:** 10.1371/journal.pone.0277171

**Published:** 2022-12-14

**Authors:** Maman Joyce Dogba, Alice Bila, Luc Sermé, Abel Bicaba, Slim Haddad

**Affiliations:** 1 Department of Family and Emergency Medicine, Faculty of Medicine, Laval University, Quebec, Canada; 2 Université Laval Research Center on Care and Front-Line Services – Pavillon Landry-Poulin, Québec, Canada; 3 Faculté de Médecine, Pavillon Ferdinand-Vandry, Université Laval, Québec, Canada; 4 Société d’Étude et de Recherche en Santé Publique, Ouagadougou, Burkina Faso; 5 Centre de Recherche du CHU de Québec – Université Laval, Québec, Canada; PLOS: Public Library of Science, UNITED KINGDOM

## Abstract

**Introduction:**

Men can play crucial roles at each stage of HIV mother-to-child-transmission (MTCT) prevention. Low male involvement in preventative MTCT (PMTCT) in Burkina Faso is partially associated with increased MTCT rates in the country. Male involvement is at the intersection of individual experiences, social locations, organizational and systemic forces. It is crucial that PMTCT interventions are co-designed with all stakeholders, using approaches which account for such interconnected elements. This study, aims to provide a deeper understanding of male involvement using an intersectionality framework.

**Methods:**

We used an intersectional theoretical approach as it positions male involvement at the intersection of social location, systemic forces, individual experiences, and dynamics within couples. We applied an interpretative qualitative description design. The study was performed at St-Camille’s hospital in Ouagadougou, Burkina Faso. Our sample was theoretical to contrast for individual experiences and socioeconomic characteristics. Eligible women were identified via chart review and invited to participate with their male partners. We conducted individual semi-structured interviews with 12 couples. We performed a semantic thematic analysis using QDA Miner to identify themes and patterns among subjective perspectives, while accounting for variations between individuals.

**Results:**

We interviewed 12 couples; 6 were serodiscordant. All women were HIV-positive. Participant ages ranged from 23 to 48 years. We found male involvement to be multidimensional and multifaceted, covering a large spectrum (from rejection to true partnership) and diverse involvement. Male involvement was limited by competing priorities, contradictory expectations, organizational opportunities and societal beliefs. We found interactions with caregivers impacted male involvement.

**Conclusion:**

This study contributed to enhancing our understanding of male involvement in PMTCT of HIV as a dynamic result of the interconnected individual, organizational and systemic experiences. Increasing male involvement will require implementation of coordinated interventions. Such interventions must strive to simultaneously integrate individual, organizational and systemic actions together.

## Background

Men can play crucial roles at each stage of HIV mother-to-child-transmission (MTCT) prevention [1]. The “man” in ‘male involvement’ refers to either the biological father or the current male partner of the pregnant woman. The “ideal” man has been portrayed as present, accessible, understanding, willing to learn more about the progress of the pregnancy, emotionally supportive of the woman (accompanying her to prenatal care, discussing breastfeeding options) [2], and/or providing financial support (paying for prenatal and postnatal care) [3, 4].

Male involvement can facilitate a woman’s access to and utilization of preventative mother-to-child transmission (PMTCT) programs [5, 6], particularly counselling services [6], secure childbirth and breastfeeding practices [7–9]. Burkina Faso, however, is far from its targeted male involvement objectives in PMTCT [10]. In fact, recent studies in Burkina Faso noticed gradual increases in residual MTCT rates from 5.30% in 2014 to 5.94%, and 8.20% in 2015 and 2016 respectively [10]. With only 2.90% of male partners screened through PMTCT programs, low male involvement in the country has been partially attributed to this trend and thus, has become a priority for more effective PMTCT in the country (Plan ETME 2017–2020) [11].

Barriers to male involvement in Burkina Faso, as is the case in many African countries, include sociocultural norms and beliefs, taboos, insufficient space dedicated to male partners in health care centers [12, 13] unfriendly care providers behavior [13, 14], fear of being informed of an HIV-positive status [14], and long waiting periods at clinics. Most interventions to increase male involvement have historically targeted women, men or care providers separately [6], in addition to gradually calling to shift the focus from women to the healthcare system. While these barriers are thought to be interconnected and may influence each other [1], most studies and programs on the topic are still addressing these barriers in an additive, rather than an interdependent, manner. Moreover, most health care interventions are designed without the input of those most impacted—the men and their partners. Therefore, it is crucial that PMTCT interventions in Africa be co-designed through collaboration with all stakeholders, including men and women, using approaches that account for interconnections between identity, social location, power imbalance and multiple forms of health discrimination [14].

By blending an understanding of structural elements with those more individually interpretative aspects of male involvement, the intersectionality framework allows health researchers and care providers to go beyond usual approaches of studying male involvement. This study, thus, aims to inform the development of such fit-for-purpose interventions to increase male involvement in PMTCT in Burkina Faso. This study, aimed to provide a deeper understanding of male involvement using an intersectionality framework.

## Methods

### Conceptual framework

This study was informed by intersectional theory as it positions male involvement at the intersection of social location, systemic forces and individual experiences, as well as highlights power dynamics within couples [14, 15]. Additionally, it focuses on the interdependence of social location and its impacts on experiences rather than applying an additive approach.

### Research design

We applied interpretive description [16] which examines a “clinical phenomenon with the goal of identifying themes and patterns among subjective perspectives, while accounting for variations between individuals” [17]. The initial study phases were oriented toward identifying the forms of male involvement whether financial and/or psychological, professional, partial or total. As patterns within the data became more apparent, we adopted an intersectional approach, which allowed us to examine the interconnections of men’s and women’s personal experiences, and the structural elements that shape their involvement.

The research team included two senior researchers (SH and AB2): the first, a specialist in health services research and impact assessment in Burkina Faso and the second, a public health specialist; two research associates (AB1 et LS): both with sociology backgrounds and a mid-career researcher (MJD) with extensive experience in qualitative health service research who was primarily involved in the data analysis. The team acknowledged multiple identities of each researcher and adopted self-reflective stances for occasional review of each researcher’s shift in attitude towards male involvement throughout the research process [18].

### Participants and setting

The study was performed at St-Camille’s hospital in Ouagadougou (henceforth referred to as the Hospital). It is a Christian hospital founded in 1967 by the Camilian’s religious community and located within the Central Health Regional Center. This not-for-profit care center also offers hospitalizations and performs complementary paraclinical examinations. It covers 9 wards (maternity, maternal and child care, laboratory, pharmacy, pediatrics, new pediatrics, neonatology, general medicine and specialized medicine, which includes 20 specialties). The maternity ward is one of the biggest within the Hospital with between 3500 and 4000 deliveries per year, 105 beds, and 4 private air-conditioned first-class rooms equipped with a television, fridge and hot running water.

The sole selection of this health facility is justified by the exploratory nature of this study as well as the excellent organization of the follow-up of people living with HIV, which facilitates research (accessible archives for example). As usual with interpretative description, our sample was theoretical. Thus, St-Camille’s hospital care providers contributed to the selection of participants, in order to contrast for individual experiences and socioeconomic characteristics. To be eligible to participate in the study, women must have i) a confirmed HIV–positive status; ii) informed her partner about her status; iii) given birth to a child at least six weeks before the study; iv) invited her partner to participate in the study. Eligible women were identified with the PMTCT care provider via chart review. A research assistant explained the study objective to the provider and assisted with chart reviews. The Hospital chart contains detailed information about the woman’s HIV status, her partner’s status and whether or not the status has been communicated to their partner.

Eligible women were invited to participate in the study at the beginning of their consultation with the care provider. Those who agreed met with the research associate after the consultation, received an in-depth explanation about the project and confirmed their willingness to participate. They were then asked to provide their consent in writing and to inform their partner about the project. Before the interview, the research associate checked the partner’s agreement to also participate and confirmed an appointment with the woman.

A total of 33 individuals were approached. This 33 ceiling is explained by the exploratory nature of this study but also the desire to limit the workload of health workers who helped with the recruitment of patients. We recruited 12 couples (12 women and 12 men). Nine individuals (3 men and 6 women) refused to participate: four women because they were either separated or threatened to be abandoned by the partners, and refused to inform their partners about the study. Two women consented to participate but were unreachable by phone.

### Data collection and analysis

One research assistant, AB1, conducted individual (one-on-one) semi-structured interviews with all of the participants between November 15^th^, 2017 and March 16^th^, 2018, using two different tailored interview grids (one for women and one for men) developed within the research team. This duration of data collection did not impact data quality or participants’ access to the health services, because of the long tradition of involvement of this health facility in research. Thus, the health facility managers ensured the usual care is not disturbed in the facility.

AB1 conducted 12 interviews in French and 12 in Mooré. The interviews took place according to participant preferences which were in a quiet room close to the clinic area (n = 14), at the participant’s home (n = 6), at their work location (n = 2) or at a restaurant (n = 2). The interviews were audio recorded and lasted an average of 59 minutes.

Promptly following each interview, field notes regarding participant reactions throughout the interview were documented in the form of written notes. Mooré interviews were simultaneously transcribed and translated into French, and French interviews were transcribed verbatim. AB1 and LS reviewed Mooré interviews recordings while verifying the verbatim transcriptions for accuracy. All interviews were anonymized and then analyzed using QDA Miner. We performed a semantic thematic analysis [19]. Two researchers (AB1, LS) developed a common codebook and independently did line-by-line transcript coding. We identified dominant categories inside the corpus as patterns became more apparent and established them as themes. Initial themes were discussed with MJD, SH and AB2, revised and reformulated as needed [19]. The analysis ended by establishing and checking relationships between the individual beliefs of the participants, the organizational structure, the systemic elements and the type of involvement, as required by an intersectional approach. The team met regularly for the purposes of analysis. Finally, we examined the male involvement trend over time, thus providing a temporal perspective of the involvement.

### Ethical approval and consent to participate

This study was approved by the Burkina Faso Health Service Research Review Board (N° 2017-12-177) and an authorization from the Minister of Health. Written informed consent was completed in person by all participants prior to commencing interviews. The study also received ethical approval from the CHU de Québec Institutional Review Board (Projet 2017–3197 / Renouvellement F9–26065).

### Inclusivity in global research

“Additional information regarding the ethical, cultural, and scientific considerations specific to inclusivity in global research is included in the Supporting Information (S1 Checklist).

## Results

The study included 12 couples (12 women and 12 men) six of which were serodiscordant. Nine couples practiced the same religion (4 Muslims, 4 Catholics and 1 Evangelic), whereas the partners of the other 3 women had religious backgrounds that differed from that of their partners. Ten of the twelve (12) couples were living together or were already traditionally married. Two couples were preparing for religion-based marriage. Women were between the ages 23 and 45 years and had between 1 and 5 children; an average of 2 children per woman. Seven of the women were undergoing their first experience with PMTCT. Five women had previous contact with PMTCT programs, as they had had 3 or 4 pregnancies since discovering their HIV-positive status. Five women were uneducated and 5 had attended primary school. Men were between the ages 24 and 48 years. Four men had primary school level education, five had attended secondary school and 3 had completed a college or university degree. Men had between 1 and 5 children with an average of 2 children per man. Other sociodemographic characteristics of the couples are summarized in Table 1 below.

**Table 1:** Participant demographics: Socio-demographic characteristics of interviewed participants.

COUPLE	AGE WOMAN (Years)	AGE MAN (Years)	AGE last child (Months)	NUMBER OF CHILDREN	HIV STATUS OF PARTNER	EDUCATION LEVEL WOMAN/ MAN	MARITAL STATUS
1	30	33	3	2	HIV (-)	Secondary/College	Divorced
2	29	32	3	2	HIV (+)	Secondary/College	Married
3	39	44	6	3	HIV (+)	None/Primary	Married
4	23	28	6	1	HIV (+)	Secondary/Secondary	Married
5	28	37	7	2	HIV (+)	Secondary/Secondary	Married
6	25	41	9	3	HIV (-)	None/Secondary	Married
7	40	48	8	5	HIV (-)	None/Primary	Married
8	32	33	8	3	HIV (-)	None/Secondary	Married
9	31	30	4	1	HIV (+)	Secondary/Secondary	Married
10	45	43	10	5	HIV (+)	Secondary/Secondary	Married
11	36	40	3	4	HIV (-)	Primary/Primary	Married
12	23	24	9	1	HIV (-)	None/Primary	(Man abandoned the home)

Regarding their HIV-related status, 6 women were informed they were HIV–positive during pregnancy, while 6 discovered it prior to the current pregnancy (2 by voluntary screening, 2 following the partners’ positive diagnosis, 1 following the woman’s illness and 1 following the child’s positive diagnosis). Five men were screened during their wives’ pregnancies, 3 following an episode of illness, 2 after their children’s positive diagnosis, 1 after his wife’s positive diagnosis and 1 following his wife’s voluntary screening. The duration of the couples’ follow-ups since receiving their status ranged between 1 and 14 years.

The thematic analysis allowed to identify four main themes describing male involvement in PMTCT: i) men’s attitudes regarding the discovery of their partner’s HIV–positive status; ii) highlights of male involvement; iii) spheres of involvement; iv) constraints to involvement. These four themes are described below with illustrative quotes.

### Theme 1: Men’s attitudes regarding the discovery of their partner’s HIV—positive status

We will first report the dynamics within the couples interviewed prior to the discovery of the HIV–positive status, then precise the moment of its discovery and end with the changes induced by the HIV–positive status on the couples’ daily lives, specifically in men’s attitudes. Most couples reported facing usual “couples’ challenges” such as temporary tensions regarding children’s education or financial difficulties prior to the discovery of their HIV—status. Nine women found it extremely difficult to inform their partners about their status. Most of them did so immediately after visiting the healthcare unit, while some needed to wait for a more appropriate moment and others needed support from healthcare providers or an elder in the family to inform their partners. Three women did not report any challenge at this phase as they did not encounter any: both partners were looking for an HIV–positive partner (couple 2), or the women denied their status; one for six months (couple 6) and another for almost 5 years (couple 5) until it became obvious that the children were sick.

Many negative reactions were reported: i) women reported being insulted and accused of being accountable for contracting HIV (2 women whose partners were both HIV- negative) or, ii) the woman reported being abandoned by her partner, who left the home without any support (1 case). Men’s positive reactions to the diagnosis were also reported by five women who felt very supported as their partners accompanied them to the health center for their own screening. Other changes indicated: i) modified sexual lifestyle (less frequent intercourse, use of condoms) either to protect the HIV–negative partner, or due to increased fatigue or occasional infections; and/or ii) more frequent discussions about childbirth or babies’ nutritional issues.

In short, men’s attitudes can be seen as a continuum ranging from violence and rejection toward an infected partner (couples 6, 1) through non-involvement or abandonment of the home (couple 12), to a true partnership and unprecedented support as a result of the HIV–infected status (couple 2, 4, 7, 9, 11).

(Male partner): When I told her to leave [the house], that freely given to me to live in, she said I cannot make her leave the house. I said there is no problem. You have the *disease*; I do not have the *disease*. I will not sit down for you to infect me. I moved out of the home. (…) she and I are no longer together it’s been about four to five months.

- Couple 12.

“Nothing has changed since discovering the *infection*. Since 2004 until now, nothing has changed. The deliveries of my three children have been by cesarean but he is still there. He helps me in everything I do, he helps me a lot. He helps raise my children as well. For example, if I need to come to weigh my child in the morning like this, he gets up, he washes the last one and take them to kindergarten. Myself as well I prepare myself to come weigh my child. It is like this that we do at home (…) it helps me a lot. He has no problem, he does not yell at me, he does not reproach me, he does not complain that I am sick no-no. So, he helps me a lot.”

- Woman 11

Male involvement usually included occasional discussions with the woman regarding her health, breastfeeding, and the child or children’s treatment. In other cases, it referred to the couple standing together to face family pressure (e.g. family asking the couple to have more children). When violence was reported, it was either verbal or physical and manifested through insults, accusations of the women for being unfaithful, and disclosure of the partner’s HIV–positive status to their friends and families.

“He went to his family to tell them that I had an illness. In that moment, I left to see the wife of his older brother who is in Saaba. I went there, they called him and he came. He said that I am coming with medicine in boxes that I am about to swallow (ingest).”

- Woman 12

Other times, the rejection was emotional with the man discontinuing any sexual intercourse. He was indifferent towards his partner or stopped financial support.

“Yes, stop all sexual relations. Even sexual relations using protection. We will no longer have sexual relations. Yes because of that (the infection). If I am not careful, she will contaminate me.”

- Partner 11

It is worth mentioning that all men who abandoned the home were also abusive either verbally or physically. It is also important to note that true partnership including discussion about every situation, as well as, financial and psychological support was mentioned within many couples. However, three contexts for true partnership must be highlighted. The first one was reported by partners who believed true partnership was “God’s” prescription for marriage and must be respected (couple 7). Another couple (couple 2), both partners were looking for an HIV- positive partner and chose to develop true partnership as their union was both the only and best option to experience fatherhood. Finally, in the case of couple 11, the woman talked about true partnership although the man reported having stopped sexual intercourse with the woman.

### Theme 2: Important periods of male involvement

During the analysis, we identified many changes in male involvement over time. Some men who were initially aggressive towards their partner changed their attitudes and became more supportive. In general, three periods were crucial to these changes: at the discovery of the HIV-positive status, during pregnancy and at childbirth. For example, women in couples 6 and 8 reported positive attitudes in the male partners’ attitude during pregnancy. They attributed these changes to ‘divine intervention’ as these women said they were praying for it. Otherwise, these changes were related to the peaceful attitude of women who decided most times not to respond or retaliate to their partner’s anger.

"I tell you it has improved. He does not talk about my illness anymore. It’s the prayer there. My prayer at the church [which made him change his behavior]. I’m going to pray there regularly. [In my intentions], I ask my husband to change his behavior.”

- Woman 6

"After, he himself was discouraged and he came, he told me, ‘I am sorry. I did not want [this], but I am sorry.’ That’s how it is, he is now arranging my ARV boxes. He arranges to dispose them well.”

- Woman 8

All 10 men who financially supported their partners during the discovery of their status, continued to do so during pregnancy and after childbirth, except for two: the first one (Partner 3) was indifferent and never supportive of his partner, while the other (Partner 12) abandoned his partner after childbirth. In the first case, the man (3) was unemployed. In the second case, he (12) was reportedly verbally abusive during their partnership, emotionally rejected the woman upon discovering her status and abandoned her after childbirth; despite seeming supportive during the pregnancy. At times, verbal abuse, indifference and emotional rejection co-existed with relational support and/or involvement in domestic tasks during pregnancy and childbirth (Partner 6 and Partner 8).

### Theme 3: Spheres of involvement

Male involvement covered various aspects of life that we grouped into four: financial support, medical care, psychological support and relational support (see Table 2). Participants reported *financial support* from their partners and their children for transportation fees, parking fees or to pay for drugs, food or for any hospital-related expenses during pregnancy follow-up or childbirth. All men, except one, reported financially supporting their partners. Financial support also covered groceries for the partner and the family.

**Table 2:** Summary table of spheres of implications and illustrations.

Spheres	Illustrations
Financial	*Care*: Travel costs: fuel and parking or taxi Medical costs: consultation tickets, notebooks, medicine, exams, deliveries (childbirth delivery costs) *Nutrition*: Supplies, bags of rice, corn
Support Medical/Therapeutic	Accompaniment during visits Therapeutic compliance, follow-up of appointments
Psychological	Interest shown, moral support/encouragement Protection against stigma
Relational	Dialogue: choice of baby’s feeding method, care received during visits, condom wear, etc. Consultation, joint decisions on sexual relations and procreation Participation in housework

"I give money for fuel (…) Sometimes it’s 1000 CFA Francs or 1500 CFA Francs. There are times when it does not go, I give her 500 CFA Francs or a little more, it’s just for fuel "

- Partner 5

"The day of the delivery as well, he accompanied me to the health center and the day after the return he paid the taxi that dropped me off at home"

- Woman 7

*Medical care* whereby the man was present during hospital visits, helping in compliance to the treatment and the follow-up of appointments was reported. More than 6 out of the 12 men interviewed reported attending prenatal care, deliveries or postnatal care with their partners.

"Yes, he was there at all times. At the time of the intervention he was there all day, he spent all day because there were too many complications that day. There were too many emergencies. When they brought me to the ward, it was already 4pm. But all day he was there. It was early in the morning that he came. He stayed with me until the intervention. When I was taken out of the operating room, he was there. When you leave the ward, you are taken to the emergency room. I returned today at 6:30pm. Now it is the next day at 11:30 in the morning that I came out of the emergencies but he was still there."

- Woman 11

"Since she could not ride a motorcycle, I accompanied her. I got up at 5 in the morning, to come drop-off the notebook and each time, I am first or second. I came to deposit the notebook of the baby; each time and I am 1st or 2^nd^ in line. I put down the child’s notebook and I go to pick her up, to bring her to the health center. When I bring her, out of the health center, I have a brother who is next door, I sit there. When she finishes, she calls me and then I come to get her. That’s what I did up until three months ago."

- Partner 1

It also included helping women to regularly take their treatment. Up to 5 men said they set alarms on their partner’s phone to remind them of taking their medication. Some men reported calling their partner in addition to the alarms, as they wished to ensure their partner’s adherence to their prescribed regimen. Moreover, psychological support can include monitoring the children’s medication to prevent treatment interruption, as well as being a source of comfort when the women are distressed or discouraged whether at time of diagnosis or after.

"… At this level [for taking medication], I programmed her cellphone for 7am and 7pm so that when it rings she takes her medication."

- Partner 6

"My cell phone and my watch are set on medication schedules”

- Partner 2

"Me, I assisted her. Every day that God makes, I am there (…) The assistance is to come and talk to her and that’s what I did. Yes, cheer her up and I’m here, anyway. I never said, (…) I never insulted her. I never accused her of anything. I did not insult her in anyway. I assisted her in all aspects, taking care, everything. I did everything and everything… "

- Partner 1

*Psychological support* was also reported and included the man being interested in the daily life of his partner, supporting her morally and encouraging her but also protecting her against stigmatization. Ten men reported psychologically supporting their partners by protecting them against stigmatization and refusing to disclose her HIV-positive status to other family members.

"Yes, she brings back the drugs, to show me, the number of boxes, the duration of treatment. But what I tell her is that the situation must remain hidden [serology], you should not expose the boxes so people know and talk about it. Otherwise we risk being separated and it will be your fault. If you know how to hide, there will be no problem. I can be infected and hide it and live without a problem. But for you, if you get up to expose this and people start to say that the wife of this person has that, I knew it… me I’ll act as if I did not know, I’ll send you back. That’s all I told her.”

- Partner 6

Finally, *relational support* was also identified though less frequent than financial and psychological support. It comprised discussions within the couple around secure breastfeeding and child feeding options, involvement in domestic tasks, communication and consensual decisions about sexual intercourse.

"… it’s only us two, he does everything, even clean the house, he cleans the house, he washes dishes, he prepares. With the pregnancy now, he even wanted me to leave it altogether, that I leave the housework; I left the work, he was going to do it. Since I gave birth in October, I think that his sister, in August, had gone to see their grandmother in the village; he was asking me to leave it and he was doing almost everything. "

- Woman 9

### Theme 4: Constraints to male involvement

We identified four major constraints to male involvement that are described below.

*Constraint 1*: *Involvement was culturally grounded and aligned with prevailing societal beliefs*

Most cases of involvement were consistent with the cultural beliefs of men. As such, those who think pregnancy and childbirth are “women’s affairs” did not see an added-value of their involvement beyond usual paternal duties (e.g. financial support to the whole family). In fact, financial support was reported by 11 out of the 12 men interviewed, followed by 9 out 12 men providing support with treatment adherence. In the event the male partner did not have stable income, he became pragmatic and accepted to share financial responsibilities with the woman.

"She is the one who supports it, she is the one who supports [the household expenses]. As two, we manage because we just moved, it was she who sold her bread and other things so that as two we can meet our needs. "

- Partner 8

"Most often it is the woman who pays the expenses. Since I do not have a job, it is difficult to (…) If it coincides that I had work, I could put something aside [to prepare for the delivery]. Since the woman is doing something, even if she does not, she can support.”

- Partner 3

Some men acknowledged male involvement as a demonstration of love yet they decided to limit their involvement either due to fear of stigmatization, being labelled a “feeble man”, or because their partners would no longer be submissive to them.

"If she herself knows that I love her, she’s going to put the rope around me [dominate] (…) no, that’s not the service. It is that in life itself, one must gently, act as if one loves them even if one does not love them so that there is understanding. Otherwise, if you clearly show that you can die for the person, you risk dying quickly."

- Partner

*Constraint 2*: *Desire for more involvement can compete with other daily priorities*

Some men reported competing priorities in their daily lives which challenges their willingness to be more involved in their children’s care. Two men travelled daily because of work. They had to decide between keeping their jobs, which is also the only source of family income and being more involved with child rearing. Another competing priority was the level of the men’s involvement with their elder healthier children.

"No, no, she says often; let me go home alone, because often it is around 8 am, where I myself have to go to work. It’s she who tells me it’s ok, she’ll be able to go home alone, I say there are no worries, good! I have also tried to help her my way."

- Partner 4

"Even herself there; Mrs. I. [PMTCT manager] asked us to go to the other side, to pediatrics, we went there once, that day. I myself went to make the appointment, I waited for her there [his companion] I myself became annoyed. She did not come quickly, even though I called her, I said no, join me there. That day I had class and I had something to do, she did not come quickly.”

- Partner 1

*Constraint 3*: *There were contradictory expectations towards involvement*

Our analysis also highlighted women’s unanimous expectation of increased involvement from their partners, both financially and psychologically. However, their expectations were somehow contradictory, given their concern regarding their loss of privacy if men were allowed into their space. In fact, pre and postnatal care as they are currently organized, are seen as private spaces where women share their lived experiences. In other words, at times, more male involvement was seen as an intrusion in such spaces.

"(…) If you go back there, it’s like a family, huh. A stranger [man] who returns, they will look at him with a different eye; maybe when you go you get used to it, it is there you’re going to be looking at yourself (…) No, because of my wife, if I’m going to frustrate others, is it not best to remove myself? No, but essentially there are others who are disturbed, essentially there are others who are disturbed when they see a stranger among them. Especially a man (…) Health workers, I believe that with health workers there is no problem, it is the other patients there. Even if they are not going to talk after, with respect to the looks you yourself will feel there that there are those who are unhappy (…) So when you return they will automatically see that as bad. Oh hoo (surely), they will see that bad."

- Partner 8

"She said a man does not come back there. If we say to come and stay for her weighing, I can go back to sit down so that we can weigh her. Now if we say that these are women’s stories and that a man cannot go in there now. I do not know if it’s a secret between the women. So, you do not have to force yourself to go home."

- Partner 7

*Constraint 4*: *Participants perceived limited organizational opportunities for involvement*

Our analysis identified additional organizational drawbacks to male involvement. Individual counselling for HIV–positive persons as is presently organized, did not offer opportunities for couples counselling. Sometimes, each partner was followed-up at a different healthcare setting (couple 1), preventing them from joint meetings with care providers, where their involvement could be triggered and their questions answered.

Some participants related their degree of involvement to the quality of counselling they were exposed to. For example, a male partner reported (couple 12) being informed of his female partner’s HIV–positive status without her consent. Moreover, he said the care provider encouraged him to abandon his home.

"When he left to get the results, the old [midwife] told him that I have an illness but not to go home with me anymore [to have sex], not without a condom, to no longer have unprotected relations. I did not understand this like that. He came to ask me what is this medicine? I said that I have itching and an ulcer. He asked me if I was telling the truth, because he did not want lies. I said to him, am I the health worker? When you left, what did they tell you? That they told him that I have AIDS but they told him not to sleep with me anymore otherwise he will be infected.”—Woman 12

## Discussion

This intersectionality theory-informed study aimed to understand and portray male involvement in PMTCT programs within couples treated at the Hospital in Burkina Faso. We carried out in depth interviews with 12 couples. All women were HIV-positive but 6 couples were serodiscordant regarding their HIV status. Our analysis led to three main observations.

First, male involvement covered a continuum, from rejection and violence to true partnership with the woman, which also encompassed a large spectrum of financial, psychological or relational involvement. Previous studies confirmed this multidimensional and multifaceted aspect of male involvement. Rejection itself could be without any support to the woman, but could also manifest through the repudiation of the woman, her abandonment, or physical violence [20]. Involvement is sometimes partial, including support with avoidance of any dialogue with the woman or support with dissimulation, whereby the man financially supported the woman without her knowledge while she was treated. Our findings also confirmed that male involvement was mainly financial [21, 22], sometimes psychological [12, 21, 23] and seldom relational [24]. While financial involvement was reported by at least 10 of the 12 men in our study and may even co-exist with violence, at least 8 men mentioned emotional support and at least 5 noted relational support. Similarly, previous studies reported that up to 88% of male partners saved money to support their partners during pregnancy, while only 25 to 29% of male partners accompanied their partners to pre or postnatal care [21]. But this involvement is not always present or absent, since involvement means different things to different people. It is therefore important to clarify what is meant by "involvement" since some men say they are involved "financially" and play their role as a "man".

On one side, this multidimensional and multifaceted involvement was culturally grounded and consistent with African traditional society; whereby the man is to be the financial provider of the home [25, 26]. However, at times, men needed to be pragmatic and accept shared financial responsibilities; especially those with unstable income. Beside financial support, psychological support was many times technological, with men setting alarms for their partners. This could be a starting point for a fit-for-purpose male involvement-related intervention such as mobile health (m-health) interventions.

On the other side, this multidimensional and multifaceted involvement was culturally challenging as women encountered contradictory expectations. While most women expected increased support from their partners, they also anticipated a loss of privilege to share their experiences among women as they recently had during prenatal and postnatal care. Women opposed to male presence in these “female spaces” sometimes said it was against nature and culturally unacceptable for men to witness childbirth [21]. Nonetheless, the increased woman’s autonomy in many African countries [26] and the numerous societal changes shifting task distribution within couples from traditional to more gender-balanced [25, 26], could positively impact male involvement.

Second, male involvement was dynamic and changing overtime. Some changes were positive; men reacting angrily at their partner’s initial HIV-positive status discovery and gradually becoming more supportive during pregnancies or childbirth. Some changes were also negative with some men seeming initially supportive and gradually becoming unsupportive or even abandoning their partners. In almost all cases, the involvement varied over time and our findings identified three critical phases: the discovery of the HIV-positive status, the onset of pregnancy, and childbirth. These phases could yield emotional changes and generate a lot of existential questions for both men and women. This dynamic involvement suggests paying specific attention to couples during those phases as well as throughout long-term follow-up of PMTCT for HIV-positive women. This dynamic involvement also calls for closer monitoring over time as an initial positive reaction and involvement of a male partner could gradually change and even end in abandonment. On the contrary, initial rejection could also gradually shift to acceptance and support over time.

Third, male involvement is inherently situated at the intersection of social location, systemic forces and individual experiences, confirming the adequacy of an intersectional theory. For example, some male partners desired to be more involved psychologically by accompanying their partner to healthcare centers but were concretely limited by the organization of pre and postnatal care, with no space allotted for men. Additionally, joint sessions for couples counselling to trigger open discussions between the couple were also lacking. On the contrary, the presence of men was often perceived by women as a threat to their intimacy despite dedicated space for men being an important criterion for quality care and management of HIV-positive women [27]. Male involvement is sometimes shaped by the attitude of healthcare professionals and men seemed more present with their partners at healthcare centers when they were invited by a healthcare professional. It is thus, not surprising that one man in our study (couple 12) reported being advised to abandon his partner to avoid contracting HIV. But the sense of child and fatherhood may change the commitment of some men since fatherhood may be perceived as a factor in the development of the male personality.

In addition, the desire for increased involvement may also be limited by prevailing societal beliefs as increased involvement could be considered a sign of weakness. This could lead to stigmatization of men who accompanied their partners, as these men are usually mocked and referred to as ‘bewitched or dominated by their wives’ [27]. Thus, interventions for more male involvement need to go beyond solely the aforementioned focus ‘shift’ onto the healthcare system [3, 28] and instead strive to simultaneously integrate individual, organizational and systemic actions (including community mobilization) together [28].

The use of an intersectional theory, in addition to the temporal perspective of the male involvement trend, is one of the strengths of our study. However, the small number of participants in the study could limit the transferability of our conclusions to our context. Despite this limitation, our findings lead to scientific and practical implications due to the detailed description of our context and the depth of our analysis, ensuring rigorous conclusions. On a scientific level, this study contributed to enhancing our understanding of male involvement in PMTCT of HIV as a dynamic result of the interconnected individual, organizational and systemic experiences, changing overtime and particularly during critical phases (discovery of status, pregnancy and childbirth). It also unveils unanswered questions that further research could address. For example, it would be interesting to examine the changes in men’s roles and norms regarding their involvement in PMTCT of HIV within the constantly shifting socio-economic context of Africa, where women are sometimes the main financial provider of the family.

On a practical level, this study helps to identify practical levers to improve male involvement. One of these levers is to promote and help maintain current involvement (mainly financial and technological). Another lever is to create friendly spaces at healthcare centers for couples counselling. While this requires long-term and well-planned actions which may also be costly, immediate zero cost interventions such as changing the attitudes of health professionals could be an effective place to start. However, it is worth mentioning that the results of this study were communicated to local authorities and provided solid arguments for the exploration of the perspectives of health workers and policy-decision makers on this topic.

## Conclusion

In conclusion, this study contributes to the understanding of the involvement of men in PMTCT of HIV. Male involvement in PMTCT of HIV is known to positively impact women and children’s health. However, this involvement is at the intersection of individual experiences, social locations, organizational and systemic forces. Therefore, continuing and improving male involvement will require implementation of coordinated and simultaneous interventions at all levels.

## Supporting information

S1 File (PDF)

S1 Checklist Inclusivity in global research. (DOCX)

## Abbreviations

HIV: human immunodeficiency virus
MTCT: Mother to child transmission
PMTCT: Preventative mother to child transmission
