# Supplementary material for: Towards a deeper understanding of male involvement in the prevention of mother to child transmission of HIV in the Bogodogo District of the Central Region of Burkina Faso
Source: PLoS One. 2022 Dec 14;17(12):e0277171. doi: 10.1371/journal.pone.0277171 (PMC9749984; doi:10.1371/journal.pone.0277171)
Supplement: S1 File — (PDF) [file pone.0277171.s001.pdf]

Projet de recherche sur l'implication des hommes dans la prévention de la transmission mère-enfant (PTME) au Burkina Faso :

Guide d'entretien des femmes séropositives ayant accouché et dont les conjoints sont au courant de la séropositivité.

Enregistrer le consentement verbal (audio) ou signer le contentement écrit

Bienvenue et Introduction : Démarrer l'enregistreuse

Merci d'avoir accepté de participer à cette entrevue. Je m'appelle Alice Bila. Je suis une professionnelle de recherche au sein de l'équipe de projet. J'aimerais aujourd'hui qu'on parle de l'implication des hommes dans la PTME.

Je vais commencer par vous poser des questions d'ordre sociodémographique. Par la suite, nous allons de votre histoire, depuis la découverte de la séropositivité jusqu'à ce jour, en insistant sur la dynamique avec votre conjoint.

Notez qu'il n'y a ni bonne ni mauvaise réponse.

Numéro de la participante : ___________________________________

1. Quel âge avez-vous ?
2. Quelle est votre religion ?
3. Où habitez-vous ? (Précisez la ville, la commune et le quartier)
4. Parlez-moi de votre situation matrimoniale : (Précisez si la femme est dans un foyer monogame ou polygame, préciser combien de femmes le mari a)
5. Avez-vous des enfants ? Si oui combien et quels âges ont vos enfants ?
6. Que faites-vous comme travail ? Et votre mari ?
7. Quelles sont les principales sources financières de la famille :
8. Quel est votre niveau d'instruction ? Qu'en est-il de votre mari ?

9. Quand et comment avez-vous découvert votre séropositivité ?
10. Comment votre mari a appris votre sérologie ? Comment a-t- réagit ?
11. Quel est le statut sérologique de votre mari ?
12. Quand est-ce qu'il a fait son test de dépistage ? (Avant ou après la femme ?)

13. Comment avez-vous été informé du statut de votre mari ? (Explorez la prise de traitement du conjoint)
14. À quel moment de la grossesse avez-vous commencé la consultation prénatale CPN (nombre d’enfants sous PTME) ?
15. Comment ça se passe avec votre mari lorsque vous devez vous rendre à la CPN ?
16. Comment ça se passe avec votre mari lorsque vous revenez de la CPN ? Comment a-t-il réagi ?
17. Depuis que vous avez connu votre sérologie, qu’est-ce qui a changée ou pas dans vos relations avec votre mari ? (intimité, rapport sexuel, changement de comportement ou pas, soutien moral, désir de procréation future)
18. Comment se comporte/réagit votre mari depuis que vous prenez votre traitement ?
19. Quelles sont les mesures que vous avez prises pour votre accouchement et pour l’alimentation de l’enfant ? Qu’en pense votre mari ? (accouchement sécurisé, et prophylaxie post natal).
20. Avez-vous des questions à nous poser ?

Conclusion : Nous sommes à la fin de notre entrevue et je vous remercie de votre participation à cette entrevue.

Projet de recherche sur l'implication des hommes dans la prévention de la transmission mère-enfant (PTME) au Burkina Faso :

Guide d'entretien des conjoints des femmes séropositives ayant accouché et ayant partagé leur séropositivité avec les conjoints

----

Enregistrer le consentement verbal (audio) ou signer le contentement écrit

Bienvenue et Introduction : Démarrer l'enregistreuse

Merci d'avoir accepté de participer à cette entrevue. Je m'appelle Alice Bila. Je suis une professionnelle de recherche au sein de l'équipe de projet. J'aimerais aujourd'hui qu'on parle de l'implication des hommes dans la PTME.

Je vais commencer par vous poser des questions d'ordre sociodémographique. Par la suite, nous allons de votre histoire, depuis la découverte de la séropositivité jusqu'à ce jour, en insistant sur la dynamique avec votre conjoint.

Notez qu'il n'y a ni bonne ni mauvaise réponse.

Numéro du participant : ___________________________________

1. Quel âge avez-vous ?
2. Quelle est votre religion ?
3. Où habitez-vous ? (Précisez la ville, la commune et le quartier). Vivez-vous avec une ou plusieurs de vos femmes ? Laquelle ou lesquelles ?
4. Parlez-moi de votre situation matrimoniale : (êtes-vous monogame ou polygame, préciser combien de femmes le mari a)
5. Avez-vous des enfants ? Si oui combien et quels âges ont vos enfants ?
6. Que faites-vous comme travail ? Et votre femme ?
7. Quelles sont les principales sources financières de la famille :
8. Quel est votre niveau d'instruction ? Qu'en est-il de votre femme ?

9. Comment avez-vous découvert la séropositivité de votre femme ?
10. Comment avez-vous réagi quand vous l'avez appris ?

11. Comment avez-vous connu votre statut sérologique ? (Explorez la sérologie des autres femmes si polygame) - Qu'avez-vous fait par la suite ? (Explorer son suivi biologique et thérapeutique)
12. Comment votre femme a réagi quand elle a été informée de votre statut sérologique ?
13. Qu'est-ce qui a changé dans votre relation avec votre femme depuis que vous avez découvert sa sérologie ? (intimité, rapport sexuel, changement de comportement ou pas, soutien moral, désir de procréation future)
14. A quel moment de la grossesse votre femme a commencé les CPN ?
15. Comment réagissez-vous lorsque votre femme vous informe qu'elle se rend à la CPN ?
16. Comment réagissez-vous lorsque votre femme revient de la CPN ?
17. Quelle est votre réaction depuis que votre femme prend son traitement ?
18. Comment aidez-vous votre femme à faire son suivi biologique et à prendre son traitement ?
19. Quelles sont les mesures que vous avez prises pour l'accouchement de votre femme et pour l'alimentation de l'enfant ? (prophylaxie post-natale, suivi biologique et thérapeutique post-natal).
20. Avez-vous des questions à nous poser ?

Conclusion : Nous sommes à la fin de notre entrevue et je vous remercie de votre participation à cette entrevue.

Research project on the involvement of men in the prevention of mother-to-child transmission (PMTCT) in Burkina Faso:

Interview guide for HIV-positive women who have given birth and whose spouses are aware of their HIV status

--

Record verbal (audio) consent or sign written consent

Welcome and Introduction: Start the recorder

Thank you for agreeing to participate in this interview. My name is Alice Bila. I am a research assistant on the project team. Today I would like to talk about men's involvement in PMTCT.

I will start by asking you some socio-demographic questions. Then, we will go through your life story, from the discovery of your HIV status to the present day, with an emphasis on the dynamic with your husband.

Note that there are no right or wrong answers

Participant's reference number : ___________________________________

1. How old are you?
2. What is your religion?
3. Where do you live? (Specify city, town and neighborhood)
4. Tell me about your marital status: (Specify whether the wife is in a monogamous or polygamous household, specify how many wives the husband has)
5. Do you have any children? If so, how many and how old are your children?
6. What do you do for a living? What about your husband?
7. What are the family's main financial sources?
8. What is your level of education? What about your husband?

9. When and how did you discover your HIV status?
10. How did your husband find out about your HIV status? How did he react?
11. What is your husband's HIV status?
12. When did he get tested? (Before or after the wife?)
13. How did you find out about your husband's status? (Explore spouse's treatment uptake)
14. At what point in the pregnancy did you start prenatal care (number of children on PMTCT)?

15. How is it with your husband when you have to go to prenatal care?

16. How is it with your husband when you come back from prenatal care? How did he react?

17. Since you have known your serology, what has changed or not in your relationship with your husband? (intimacy, sexual relationship, change in behavior or not, moral support, desire for future procreation)

18. How has your husband behaved/reacted since you started taking your treatment?

19. What measures have you taken for your delivery and for the child's feeding? What does your husband think about it? (safe delivery, and post-natal prophylaxis).

20. Do you have any questions for us?

We are at the end of our interview, and I thank you for your participation in this interview.

Research project on the involvement of men in the prevention of mother-to-child transmission (PMTCT) in Burkina Faso:

Interview guide for spouses of HIV-positive women who have given birth and shared their HIV status with spouses

---

Record verbal (audio) consent or sign written consent

Welcome and Introduction: Start the recorder

Thank you for agreeing to participate in this interview. My name is Alice Bila. I am a research professional on the project team. Today I would like to talk about men's involvement in PMTCT.

I will start by asking you some socio-demographic questions. Then, we will go through your story, from the discovery of your HIV status to the present day, with an emphasis on the dynamic with your spouse.

Note that there are no right or wrong answers.

Participant's reference number : ___________________________________

1. How old are you?
2. What is your religion?
3. Where do you live? (Specify city, town and neighborhood). Do you live with one or more of your wives? Which one or ones?
4. Tell me about your marital status: (are you monogamous or polygamous, specify how many wives the husband has)
5. Do you have any children? If so, how many and what ages are your children?
6. What do you do for a living? And your wife?
7. What are the family's main financial sources?
8. What is your level of education? What about your wife?

9. How did you find out about your wife's HIV status?
10. How did you react when you found out?
11. How did you find out your HIV status? (Explore other women's HIV status if polygamous) - What did you do afterwards? (Explore her biological and therapeutic follow-up)
12. How did your wife react when she was informed of your HIV status?

13. What has changed in your relationship with your wife since you found out about her HIV status (intimacy, sexual relationship, change in behavior or not, moral support, desire for future reproduction)

14. At what point in the pregnancy did your wife start ANC?

15. How do you react when your wife informs you that she is going to ANC?

16. How do you feel when your wife returns from ANC?

17. What is your reaction since your wife started taking her treatment?

18. How do you help your wife with her biological monitoring and treatment?

19. What measures have you taken for your wife's delivery and for the child's feeding (post-natal prophylaxis, biological monitoring, and post-natal therapy)?

20. Do you have any questions for us?

We are at the end of our interview and I would like to thank you for your participation in this interview.
